# Supplementary material for: Symptoms of depression in a large healthy population cohort are related to subjective memory complaints and memory performance in negative contexts
Source: Psychol Med. 2017 Jun 19;48(1):104–14. doi: 10.1017/S0033291717001519 (PMC5729845; doi:10.1017/S0033291717001519)
Supplement: Supplementary file 1 [file S0033291717001519sup001.docx]

# Supplementary Material

# Symptoms of Depression in a Large Healthy Population Cohort are related to Subjective Memory Complaints and Memory Performance in Negative Contexts

Susanne Schweizer; Rogier A. Kievit; Tina Emery; Cam-CAN; Richard N. Henson

Index:

Supplementary Results

Table S1. Sample characteristics

Table S2. Relationship between memory and depressive symptom measures and covariates

### Supplementary results: Moderating effect of gender on the association between depressive symptoms and hippocampal volume

There was no moderating effect of sex on the association between depressive symptoms and hippocampal volume (adjusted for total intracranial volume), *β* = 2.40^–5^, *SD* = 2.55^–5^, 95%CI [–2.62^–5^; 7.41^–5^], *t*(587) = 0.94, *p* = .348, *R^2^*= .43, *R^2^_adj_* = .43.

### Table S1. Sample characteristics

|  | Overall cohort | Neuroimaging cohort | Affective cohort |
| --- | --- | --- | --- |
|  | *N* = 2544 | *n* = 592 | *n* = 288 |
|  | *M* (*sd*); range |  |  |
|  | Skewness; Kurtosis |  |  |
|  | For binary variables: *N* (%) |  |  |
| Demographics |  |  |  |
| Age | 59.48 (20.75); 18-98 | 54.04 (18.50); 18-88 | 53.68 (18.00); 18-87 |
|  | – 0.23; – 1.22 | – 0.07; – 1.14 | – 0.05; – 1.06 |
| Female | 1427 (56) | 302 (51) | 153 (50) |
|  |  |  |  |
| Memory measures |  |  |  |
| Standard memory (Recall) | 11.47 (4.92); 0-24 | 12.99 (4.29); 0-24 | 13.02 (4.01); 2-22 |
|  | – 0.26; – 0.35 | – 0.32; – 0.28 | – 0.35; – 0.24 |
| Subjective memory complaints (Yes) | 959 (38) | 181 (31) | 93 (32) |
|  |  |  |  |
| Number of subjective memory complaints | 5.67 (7.78); 0-34 | 4.49 (7.09); 0-31 | 4.78 (7.27); 0-28 |
| (in those reporting memory complaints) | 0.94; – 0.39 | 1.15; – 0.11 | 1.07; – 0.33 |
| Object recall in *neutral* contexts |  |  | .74 (.17); .10-.98 |
|  |  |  | – .92; .55 |
| Object recall in *negative* contexts |  |  | .76 (.17); .20-1.00 |
|  |  |  | – .94; .44 |
| Object recall in *positive* contexts |  |  | .77 (.16); .15-1.00 |
|  |  |  | – 1.27; 1.55 |
| Clinical measures |  |  |  |
| Symptoms of depression | 3.28 (2.89); 0-17 | 2.80 (2.55); 0-17 | 2.79 (2.32); 0-13 |
|  | 1.33; 2.07 | 1.41; 2.69 | 1.29; 2.36 |
| Symptoms of anxiety | 5.16 (3.38); 0-20 | 5.01 (3.30); 0-20 | 5.15 (3.10); 0-17 |
|  | 0.82; 0.70 | .91; 1.18 | .85; .95 |
| Covariate measure |  |  |  |
| Cognitive ability | 51.23 (5.09); 14-56 | 31.03 (8.28); 0-44 | 31.11 (7.73); 0-44 |
|  | – 2.12; 7.22 | – 1.42; 3.07 | – 1.13; 2.25 |

*Table S1* Age = age in years; Object recall = proportion of objects recalled correctly; symptoms of depression = total score on the depression subscale of the Hospital Anxiety and Depression Scale (HADS); symptoms of anxiety = total score on the anxiety subscale of the HADS; Cognitive ability = for the overall cohort it was assessed with the total score on the Addenbrookes Cognitive Examination Revised without the memory subscale; for the neuroimaging and affective cohorts is was assessed using the Cattell culture-free intelligence test.

### Table S2. Relationship between memory and depressive symptom measures and covariates

|  | Age | Cognitive ability^a^ | Sex |
| --- | --- | --- | --- |
|  | *r* | *r* | *r*_biserial_/Χ^2^ |
| Overall cohort (*N* = 2544) |  |  |  |
| Standard memory (Recall) | –.47*** | .45*** | .13** |
| Subjective memory complaints | .41*** | –.32*** | .07 |
| Symptoms of depression  Neuroimaging cohort (*n* = 592) | .20*** | –.14** | .00 |
| Standard memory (Recall) | –.39*** | .47*** | .19** |
| Subjective memory complaints | .29*** | –.21*** | –.01 |
| Symptoms of depression | .10* | –.14*** | –.01 |
| Affective cohort (*n* = 288) |  |  |  |
| Standard memory (Recall) | –.30*** | .42*** | .23*** |
| Subjective memory complaints | .21*** | –.14** | .02 |
| Object recall in *neutral* contexts | –.55*** | .51*** | .17** |
| Object recall in *negative* contexts | –.56*** | .48*** | .24*** |
| Object recall in *positive* contexts | –.53*** | .49*** | .24*** |
| Symptoms of depression | .11 | –.16** | .02 |

*Table S2 r* = Pearson’s correlation coefficient; *r*_biserial_/Χ^2^ = biserial correlations between dichotomous and continuous variables and Chi-square test for two dichotomous variables (i.e., subjective memory complaints and sex). ^a^ ACE-R without memory subscale for the overall cohort and Cattel culture-free test for the neuroimaging and affective subsamples. ** *p* < .05; ** *p* < .01; *** *p* < .001.
